# A cross-sectional study of global DNA methylation and risk of colorectal adenoma

**DOI:** 10.1186/1471-2407-14-488

**Published:** 2014-07-07

**Authors:** Will D King, Janet E Ashbury, Sherryl A Taylor, M Yat Tse, Stephen C Pang, Jacob A Louw, Stephen J Vanner

**Affiliations:** 1 Department of Public Health Sciences, Queen’s University, Kingston, ON, Canada; 2 Department of Medical Genetics, University of Alberta, Edmonton, AB, Canada; 3 Molecular Diagnostics, Genetic Laboratory Services, Alberta Health Services, Edmonton, AB, Canada; 4 Department of Biomedical and Molecular Sciences, Queen’s University, Kingston, ON, Canada; 5 Department of Medicine, Division of Gastroenterology, Hotel Dieu Hospital/Queen’s University, Kingston, ON, Canada; 6 Gastrointestinal Diseases Research Unit (GIDRU), Queen’s University, Kingston, ON, Canada

**Keywords:** Global DNA methylation, Colorectal adenoma, Colorectal cancer, LINE-1 DNA methylation

## Abstract

**Background:**

The methylation of DNA is recognized as a key epigenetic mechanism and evidence for its role in the development of several malignancies is accumulating. We evaluated the relationship between global methylation in DNA derived from normal appearing colon mucosal tissue and blood leukocytes, and colorectal adenoma risk.

**Methods:**

Patients, aged 40 to 65, scheduled for a screening colonoscopy were recruited. During the colonoscopy, two pinch biopsies of healthy, normal appearing mucosa were obtained from the descending colon. A fasting blood sample was also collected. The methylation status of LINE-1 (long interspersed nuclear element-1) repetitive sequences, as a surrogate measure of global methylation, was quantified in DNA extracted from normal colon mucosa and blood leukocytes. Statistical analysis of the relationship between global DNA methylation and adenoma risk was conducted on 317 participants, 108 subjects with at least one pathologically confirmed adenoma and 209 subjects with a normal colonoscopy.

**Results:**

A statistically significant inverse relationship was observed between LINE-1 methylation in colon tissue DNA and adenoma risk for males and for both sexes combined for the lowest methylation quartile compared to the highest (adjusted ORs = 2.94 and 2.26 respectively). For blood, although the overall pattern of odds ratio estimates was towards an increase in risk for lower methylation quartiles compared to the highest methylation quartile, there were no statistically significant relationships observed. A moderate correlation was found between LINE-1 methylation levels measured in tissue and blood (Pearson correlation 0.36).

**Conclusions:**

We observed that lower levels of LINE-1 DNA methylation in normal appearing background colon mucosa were associated with increased adenoma risk for males, and for both sexes combined. Though these findings provide some support for a relationship between LINE-1 DNA methylation in colon mucosal tissue and adenoma risk, large prospective cohort studies are needed to confirm results. Until such investigations are done, the clinical usefulness of LINE-1 methylation as a biomarker of increased adenoma risk is uncertain. Regardless, this study contributes to a better understanding of the role of global DNA methylation as an early event in CR carcinogenesis with implications for future etiologic research.

## Background

There is wide-spread interest in clarifying the role of aberrant global DNA methylation as an early event in colorectal carcinogenesis. Colorectal cancer, the third most commonly diagnosed cancer worldwide, is associated with significant mortality and morbidity [1-3]. Despite decreasing trends in colorectal cancer mortality rates over the last 10 years in both males and females, colorectal cancer remains the second most common cause of cancer death in Canada and the United States for both sexes combined [4,5]. The classical model for colorectal tumour development involves a stepwise histological progression from aberrant proliferative epithelial dysplasia to adenoma (adenomatous polyp) to colorectal cancer (adenocarcinoma) [6-9].

The methylation of DNA is recognized as a key epigenetic mechanism in the regulation of gene expression and chromosomal stability, and evidence for its role in the development of several malignancies is accumulating [10-12]. Global DNA methylation refers to the overall genome-wide content of methylated cytosines within CpG (cytosine-phosphate-guanine) sites [13,14]. The majority of CpG sites (about 80%) are found in repetitive sequences, multiple copies of DNA that are normally methylated [15]. LINE-1 (long interspersed nuclear element-1) sequences, with an average size of 900 base pairs, comprise approximately 17% of the human genome [16] and are the most widely studied repetitive sequence within the context of global DNA methylation measurement [17,18]. LINE-1 methylation levels have been shown to represent a reliable surrogate measure of global DNA methylation [19-23].

Global DNA hypomethylation, which is characterized by a generalized decrease in the number of methylated cytosines within CpG sites, is recognized as an early and consistent event in colorectal carcinogenesis [24-28] and is associated with mechanisms that drive the early stages of the carcinogenic process including chromosomal instability [29-31], elevated chromosomal mutation rates [32,33] and loss of imprinting [34-36]. Yamada et al. [28] observed a significantly increased number of microadenomas (small colonic intramucosal lesions) in hypomethylated mouse models as compared to controls suggesting that hypomethylation may promote early stage tumour development in the colon in mice [28]. In humans, the role of global DNA methylation in colorectal tumourigenesis has primarily been investigated by comparing methylation patterns in colorectal tumour tissue, with matched adjacent normal appearing tissue obtained from the same patient [37-42] or with normal colon tissue from non-diseased control subjects [37,38]. These studies indicate that virtually all colorectal tumours (benign adenomas and cancers) display a higher degree of reduction in methylated cytosines within CpG sites (global hypomethylation) as compared to matched and unmatched normal appearing colon tissue. However, from a carcinogenic mechanism perspective, these study designs using tumour tissue are limited with respect to distinguishing global hypomethylation occurring early in the dysplasia-adenoma sequence that may drive tumour initiation, from methylation changes occurring later in the adenoma-carcinoma sequence, or those which may promote tumour progression or simply be passengers in the process [43].

Contrary to this approach using tumour tissue, a study design that compares global methylation patterns in normal colon mucosal biopsy tissue from colonoscopy patients with colorectal adenomas (as surrogate end points for colorectal cancer) to participants without adenomas has the potential to better elucidate the role of aberrant global methylation as a potential marker of the early stages of colorectal adenoma/cancer etiology. Only two small previous observational studies by Cravo et al. [44] (N = 12 adenoma, N = 8 normal colonoscopy) and Pufulete et al. [45] (N = 35 adenoma, N = 76 normal colonoscopy) have examined the relationship between global methylation levels and colorectal adenoma using this type of study design [44,45]. Results indicating lower global methylation levels in normal appearing healthy colorectal mucosa of patients with colorectal adenomas [44,45] as compared to patients without colorectal pathology support the premise that aberrant global hypomethylation may represent a pervasive ‘field’ change throughout the colorectal mucosa that may precede and/or initiate the development of colorectal neoplasia/adenoma [40,42,46].

To better understand the role of aberrant global DNA methylation in the earliest stages of colorectal tumourigenesis (adenoma development) at the population level, further assessment of the association between global methylation and adenoma risk is warranted. To this end, we evaluated the relationship between LINE-1 methylation levels (as a surrogate measure of global methylation) in normal appearing colon mucosal tissue samples and adenoma risk within a large healthy screening colonoscopy patient population. We hypothesized that aberrant global methylation in normal appearing colon mucosa would reflect an underlying predisposition to the development of colorectal adenomas and therefore, global DNA hypomethylation would be associated with an increased risk of adenomas. We also investigated the association between LINE-1 methylation levels measured in blood leukocytes and adenoma risk as a secondary objective.

## Methods

### Study population

Patients aged 40 to 65 scheduled for a screening colonoscopy at a regional endoscopy centre at Hotel Dieu Hospital in Kingston, Ontario, between 2009 and 2012 were recruited by mail approximately 1–4 months prior to their colonoscopic procedure. Indications for colonoscopy included a positive family history of colorectal adenoma or cancer in a first or second degree relative, a positive fecal occult blood test (FOBT) result and average risk screening. Patients with previously diagnosed inflammatory bowel disease (IBD -ulcerative colitis or Crohn’s disease), and patients with a known history of genetic disorders that predispose to colorectal cancer (hereditary nonpolyposis colorectal cancer, familial adenomatous polyposis) were not recruited for this study. Subjects with any GI abnormality (adenoma, hyperplastic polyp or cancer) detected at a previous colonoscopy or with a new diagnosis, recurrence or treatment of any cancer type (except non-melanoma skin cancer) in the 5 years prior to colonoscopy were also not invited to participate in the study. In addition, those diagnosed with IBD or colorectal cancer based on current colonoscopy findings were excluded from the final study population as it was a concern that global DNA methylation levels in these patients may not be consistent with the target time period of interest (pre-adenoma development). In order to define a homogeneous adenoma outcome group, patients with serrated adenomas, sessile serrated adenomas or only hyperplastic polyp(s), were not included in the analysis.

At the colonoscopy visit, a fasting venous blood sample was collected in an EDTA (ethylenediaminetetraacetic acid) vacutainer which was immediately placed on ice, and centrifuged within 45 minutes of the blood draw. The buffy layer (blood leukocytes) was removed and stored at -20ºC until DNA extraction. During the colonoscopy, in an attempt to represent overall methylation levels in the descending colon, two pinch biopsies of healthy, normal appearing mucosa were obtained from the descending colon, 10 cm apart, and at least 10 cm away from any lesion, polyp or other mucosal abnormality. Specimens were immediately placed in cell lysis solution (5-PRIME DNA Isolation kit, Inter Medico, Markham, ON, Canada) and stored at -20°C.

### Laboratory methods

DNA was extracted from colon mucosal tissue biopsies and blood leukocytes using the 5-PRIME DNA isolation kit (Inter Medico, Markham, ON, Canada) according to instructions provided by the manufacturer and purified DNA was stored at −20°C until use. High-resolution melting (HRM) profile analysis, a real-time florescence-based polymerase chain reaction method, was used to measure methylation status of LINE-1 repetitive sequences, as a surrogate measure of global DNA methylation, in DNA extracted from each colon tissue sample and blood leukocytes. The application and validation of HRM to the measurement of LINE-1 DNA methylation has been described in detail previously [47]. Briefly, prior to HRM, DNA was bisulfite-converted leading to a primary sequence change in the DNA that permits differentiation of unmethylated cytosines from 5-methyl-cytosine [48]. Primers were designed to target the LINE-1 consensus promoter region, from which 8 representative CpG sites of interest, previously validated as representative of global DNA methylation status, were selected for HRM profile analysis [20]. A LINE-1 loci-specific percent methylated value for this representative sub-set of CpG sites was obtained by comparing melting curves of participant bisulfite-converted DNA to a set of standard DNA reference controls with known levels of unmethylated and methylated cytosines. LINE-1 methylation analysis was carried out in triplicate for bisulfite-converted DNA from each of the two tissue DNA samples and from blood leukocytes on 96-well plates. Each plate also included a no-template control, a set of reference methylation standards, and three replicates of internal control peripheral blood DNA to allow for assessment of inter-assay variability.

Triplicate measures of LINE-1 methylation were obtained for each of the two tissue DNA samples and blood leukocyte DNA. Individual triplicate measures were excluded where PCR values were not satisfactory due to a high PCR threshold crossing point (Cp value > 27). In addition, individual outliers (defined as >10% difference from each of the remaining triplicate values) were excluded from the analysis [47]. Average tissue and blood leukocyte percent methylation values for each individual subject were calculated by averaging all remaining triplicates for the two tissue DNA samples and averaging the remaining triplicates for the blood DNA samples respectively.

### Statistical analysis

To determine whether LINE-1 methylation levels in colon tissue or blood leukocyte DNA differ between patients with and without adenomas, outcome status was defined as follows: Study subjects with no abnormality detected at colonoscopy were assigned to the ‘normal colonoscopy’ group. Any abnormal tissue removed during colonoscopy was assessed by an expert gastrointestinal pathologist using standard histologic criteria as per hospital procedures. Using standard diagnostic criteria, subjects with one or more pathologically-confirmed tubular, tubulo-villous or villous adenoma(s) comprised the adenoma group.

Overall average LINE-1 methylation levels for colon tissue and blood leukocyte DNA were categorized into quartiles based on sex-specific distributions among participants with a normal colonoscopy. Adenoma risk was also examined using a continuous representation of sex-specific standardized LINE-1 methylation measures. These analyses were conducted separately for males and females, and for both sexes combined. Unconditional logistic regression was performed to estimate odds ratios (OR) and 95% confidence intervals (CI) as measures of association controlling for age. Sex-specific methylation measures negated the necessity to further control for sex and other risk factors for adenoma were not considered as potential covariates as they were hypothesized to be on the causal pathway. The odds ratio provides a measure of direction, strength and statistical significance of the relationships of interest – but does not estimate the prevalence ratio in this study sample.

Institutional ethics approval was obtained from the Queen’s University Health Sciences and Affiliated Teaching Hospitals Research Ethics Board (File No. 6004521) and all subjects provided informed consent.

## Results

Of the 728 subjects who met the initial eligibility criteria and were mailed recruitment packages, 444 consented to participate in the study (61% response rate). A further 48 subjects were excluded due to colonoscopy scheduling problems, or because the colonoscopy was incomplete (the examination did not reach the cecum) or the attending clinician did not obtain the two healthy tissue biopsies. Of the 396 remaining subjects, 66 were excluded on the basis of an ineligible diagnosis resulting from the colonoscopy (IBD, colorectal cancer, serrated adenoma, sessile serrated adenoma or hyperplastic polyps) leaving 330 subjects eligible for tissue and blood methylation measurement. DNA methylation was successfully measured in tissue for 317 subjects, and of these, 280 also had blood methylation measures. Pathology results for the tissue methylation analysis (n = 317) identified 108 participants with at least one adenoma (101 with tubular adenomas and 7 with tubulo-villous adenomas) and 209 subjects with a normal colonoscopy.

HRM was performed in triplicate on DNA isolated from each tissue and blood sample. The intra-assay coefficient of variation within tissue and blood triplicates was 1.84% and 1.81% respectively. An internal control sample was included on each of the 37 plates and the inter-assay coefficient of variation was 0.89%. Two colon tissue samples with completed methylation measures were available for 262 participants (Pearson correlation between the averages of the two tissue samples = 0.66) and methylation values were available for one tissue sample for the remaining 55 study subjects.

The primary indication for colonoscopy in our patient population was a positive family history for colorectal cancer or colorectal adenoma (Table 1). Those undergoing a colonoscopy because of a positive fecal occult blood test (FOBT) were more likely to be diagnosed with adenoma than the positive family history group. Males were more often diagnosed with adenoma as compared to females (53% versus 20% respectively).

**Table 1:** Description of patient population

	**Normal**		**Adenoma**			
	**n**	**(%)**	**n**	**(%)**	**Odds ratio**	**(95% CI)**
Indication for colonoscopy						
Positive family history*	171	(71%)	70	(29%)	1.00	referent
Positive FOBT**	33	(51%)	32	(49%)	2.37	(1.35-4.15)
Average risk screening	5	(45%)	6	(55%)	2.93	(0.87-9.92)
Sex						
Female	144	(80%)	36	(20%)	1.00	referent
Male	65	(47%)	72	(53%)	4.43	(2.70-7.28)
Age						
40-49	39	(71%)	16	(29%)	1.00	referent
50-54	65	(66%)	33	(34%)	1.24	(0.60-2.54)
55-59	56	(65%)	30	(35%)	1.31	(0.63-2.71)
60-65	49	(63%)	29	(37%)	1.44	(0.69-3.03)

*Positive family history of cancer or adenoma.

**Positive fecal occult blood test.

LINE-1 methylation values in tissue were approximately normally distributed within males, females and both sexes combined. Table 2 presents the relationship between quartiles of LINE-1 DNA tissue methylation and adenoma risk for males, females, and both sexes combined. The highest quartile of LINE-1 methylation was used as the referent for odds ratio estimates. ORs (adjusted for age) were larger for males as compared to females, but not statistically different (p-value interaction = 0.65). Males in the lowest quartile had a statistically significant elevation in adenoma risk (age adjusted OR 2.94, 95% CI: 1.02-8.47). For both sexes combined, there was a pattern of increasing ORs from high to low methylation quartiles, and a statistically significant increase in adenoma risk for those in the lowest quartile (age and sex adjusted OR = 2.26, 95% CI: 1.11-4.58).

**Table 2:** Association between LINE-1 DNA methylation in normal colon tissue biopsies and adenoma risk (n = 317)

**LINE-1 methylation quartile**	**Normal**		**Adenoma**				Adjusted*		
	**N**	**(%)**	**N**	**(%)**	**OR**	**(95% CI)**	**OR**	**95% (CI)**	p-value**
Both sexes***									
1 Low	52	(60%)	35	(40%)	2.23	(1.10-4.51)	2.26	(1.11-4.58)	0.02
2	53	(63%)	31	(37%)	1.94	(0.95-3.95)	2.01	(0.98-4.11)	0.06
3	51	(66%)	26	(34%)	1.69	(0.81-3.51)	1.68	(0.80-3.50)	0.17
4 High	53	(77%)	16	(23%)	1.00	referent	1.00	referent	
Standardized continuous					0.87	(0.69-1.10)	0.85	(0.68-1.08)	0.19
**Males**									
1 (79.87 - 87.42)	16	(42%)	22	(58%)	2.60	(0.92-7.30)	2.94	(1.02-8.47)	0.05
2 (87.43 - 89.55)	17	(44%)	22	(46%)	2.44	(0.88-6.82)	2.85	(0.99-8.18)	0.05
3 (89.56 - 92.06)	15	(44%)	19	(56%)	2.39	(0.83-6.87)	2.52	(0.87-7.32)	0.09
4 (92.07 - 95.94)	17	(65%)	9	(35%)	1.00	referent	1.00	referent	
Standardized continuous					0.84	(0.60-1.81)	0.80	(0.56-1.14)	0.21
**Females**									
1 (75.13 - 85.59)	36	(73%)	13	(27%)	1.86	(0.66-5.19)	1.80	(0.64-5.05)	0.26
2 (85.60 - 87.86)	36	(80%)	9	(20%	1.29	(0.43-3.83)	1.27	(0.43-3.79)	0.67
3 (87.87 - 89.95)	36	(84%)	7	(16%)	1.00	(0.32-3.14)	0.98	(0.31-3.07)	0.97
4 (89.96 - 97.95)	36	(84%)	7	(16%)	1.00	referent	1.00	referent	
Standardized continuous					0.87	(0.60-1.25)	0.87	(0.60-1.25)	0.44

*Odds ratios are adjusted for age (continuous).

**Age adjusted Wald chi square p-value.

***LINE-1 methylation quartiles based on sex-specific quartiles.

Methylation values in blood were available for 280 patients (185 normal colonoscopies and 95 adenomas). A moderate correlation was observed between blood and tissue methylation values (Pearson correlation 0.36). Although the overall pattern of odds ratio estimates was towards an increase in risk for lower methylation quartiles in blood leukocytes compared to the highest methylation quartile (referent), there were no statistically significant relationships observed (Table 3).

**Table 3:** Association between LINE-1 DNA methylation in blood leukocytes and adenoma risk (n = 280)

**LINE-1 methylation quartile**	**Normal**		**Adenoma**				Adjusted*		
	**N**	**(%)**	**N**	**(%)**	**OR**	**(95% CI)**	**OR**	**95% (CI)**	p-value**
Both sexes***									
1 Low	47	(64%)	26	(36%)	1.38	(0.67-2.86)	1.39	(0.67-2.89)	0.37
2	45	(61%)	29	(39%)	1.61	(0.79-3.31)	1.67	(0.81-3.44)	0.17
3	48	(69%)	22	(31%)	1.15	(0.55-2.41)	1.13	(0.54-2.39)	0.75
4 High	45	(71%)	18	(29%)	1.00	referent	1.00	referent	
Standardized continuous					0.85	(0.66-1.09)	0.83	(0.65-1.07)	0.15
**Males**									
1 (79.87 - 87.42)	15	(45%)	18	(55%)	1.40	(0.50-3.93)	1.38	(0.49-3.89)	0.54
2 (87.43 - 89.55)	14	(39%)	22	(61%)	1.83	(0.66-5.09)	1.85	(0.67-5.17)	0.24
3 (89.56 - 92.06)	15	(52%)	14	(48%)	1.09	(0.38-3.15)	1.06	(0.37-3.08)	0.92
4 (92.07 - 95.94)	14	(54%)	12	(46%)	1.00	referent	1.00	referent	
Standardized continuous					0.72	(0.47-1.09)	0.71	(0.47-1.08)	0.11
**Females**									
1 (75.13 - 85.59)	32	(80%)	8	(20%)	1.29	(0.40-4.15)	1.36	(0.42-4.44)	0.61
2 (85.60 - 87.86)	31	(82%)	7	(18%)	1.17	(0.35-3.87)	1.27	(0.38-4.29)	0.70
3 (87.87 - 89.95)	33	(80%)	8	(20%)	1.25	(0.39-4.02)	1.28	(0.39-4.16)	0.68
4 (89.96 - 97.95)	31	(64%)	6	(16%)	1.00	referent	1.00	referent	
Standardized continuous					0.94	(0.63-1.41)	0.91	(0.61-1.38)	0.66

*Odds ratios are adjusted for age (continuous).

**Age adjusted Wald chi square p-value.

***LINE-1 methylation quartiles based on sex-specific quartiles.

Results for continuous representations of sex-specific standardized LINE-1 methylation levels in colon tissue or blood leukocytes showed no relationships with adenoma (see Table 2 and Table 3). Findings were unaffected for all analyses (tissue and blood) when the study population was restricted to subjects with a positive family history of CRC or adenoma in a first or second degree relative (data not shown).

## Discussion

This cross-sectional study utilized a healthy screening colonoscopy population to assess the relationship between LINE-1 methylation levels measured in normal appearing background colon tissue DNA (as a surrogate for global methylation), and colorectal adenoma risk. Overall, our results support a relationship between global DNA hypomethylation in normal appearing background colon mucosa and increased colorectal adenoma risk. A statistically significant inverse relationship was observed between LINE-1 methylation in tissue DNA and adenoma risk for males and for both sexes combined for the lowest methylation quartile compared to the highest (adjusted ORs = 2.94 and 2.26 respectively). The overall pattern of effects was consistent with an increased risk of adenoma for subjects in the lower methylation quartiles in comparison to those with the highest levels of methylation.

The results of our study, which is the largest study published to date that evaluated the relationship between global methylation and colorectal adenoma development by comparing LINE-1 DNA methylation levels in normal appearing colon tissue biopsies between colonoscopy patients with and without adenomas, are generally consistent with two previous smaller observational studies that assessed this relationship (studies included 12 and 35 adenoma cases respectively) [44,45]. Our study improved upon these two studies by including a larger number of participants recruited from a clinically relevant healthy screening population. In addition, our results are in keeping with Belshaw et al. [49], who reported significantly lower LINE-1 methylation levels in colonic crypts isolated from morphologically normal colorectal mucosa from colorectal cancer patients as compared to subjects with no known gastrointestinal pathology [49].

Given that colorectal adenomas are more common in males as compared to females [50,51], that sex-specific differences in risk factors for colorectal tumours have been reported [52,53], and that many studies have found that LINE-1 methylation levels are higher in males [54], assessing the relationship between LINE-1 methylation levels and adenoma risk stratified by gender is important for understanding the role of aberrant LINE-1 methylation in CRC etiology [55]. To the best of our knowledge, this is the first study to report sex-specific relationships within the context of colorectal adenoma/cancer risk, though differences in bladder cancer risk for males and females have been observed [23,56,57]. Our results indicating a relationship between global/LINE-1 hypomethylation and increased adenoma risk for males but not females suggest that aberrant global DNA methylation patterns in males may play a more significant role in early CR tumour development as compared to females. However, sex-specific results should be interpreted with some caution, as sample size was limited, in particular for the female-specific analysis.

Though our results were consistent with an elevated risk of adenoma associated with lower blood leukocyte methylation quartiles compared to the highest methylation quartile for all subjects combined and sex-specific analyses, no statistically significant relationships were observed between LINE-1 methylation levels in blood leukocytes and adenoma risk. These results are in contrast with two previous studies that both reported an increased risk of adenoma associated with lower global methylation levels measured in blood leukocytes [45,58].

We observed only a moderate correlation between LINE-1 methylation levels measured in colon tissue and blood leukocytes which may explain the null results for blood LINE-1 methylation and adenoma risk. In addition, fewer subject were available for the blood leukocyte analysis and statistical power to detect associations was therefore limited. Our non-significant findings together with only a moderate correlation between colon tissue and blood leukocyte LINE-1 methylation have important implications for planning future epidemiologic investigations of methylation markers and colorectal cancer/adenoma risk, when, due to practical considerations, only blood or other accessible sources of DNA are available for methylation analysis [59].

This clinic-based study utilizing a biologic marker of increased risk avoids or limits many traditional biases of observational studies. In particular, information bias is unlikely as this study relied on blinded methylation analysis of tissue samples and pathology reports to assess exposure and outcome respectively. Although our study population is not representative of the overall general population (due to the colonoscopy clinic-based recruitment and response rates), this is less of a concern given that our sample represents a relevant sub-group of referrals to a regional colonoscopy screening program and also, since the study objectives are oriented towards understanding a biologic relationship postulated to be consistent irrespective of the population studied. In addition, traditional risk factors are thought to be upstream of DNA methylation (on the causal pathway) such that control for confounding is not a concern.

This study relied on cross-sectional exposure and outcome measures, and was therefore subject to the classical limitations of cross-sectional design with respect to temporality, prevalent events, and representation of exposure windows. However, each of these limitations was mitigated to some degree by the examination of exposure (LINE-1 methylation) and outcome (adenoma) that are themselves intermediate events in a causal chain, with a shorter temporal scale than exposure-cancer relationships. However, reverse causality, remains a potential explanation for the relationship observed.

Non-differential misclassification of both outcome (adenoma) and exposure (LINE-1 methylation) may have biased our results towards the null. The ‘miss rate’ of colonoscopy for adenomas has been reported to be from 6-12% for adenomas one centimetre or larger and up to 25% for adenomas less than one centimetre in diameter [60]. For our study, it is expected that the rate of missed adenomas is of less concern as all colonoscopies were performed at a single clinical centre by experienced academic gastroenterologists who each perform more than 200 colonoscopies per year and participate in an ongoing quality control program with specific audited goals for polyp detection and cecal intubation rates [61,62].

Our cross-sectional measure of global DNA methylation is intended to represent LINE-1 methylation levels prior to the initiation of the dysplasia-adenoma sequence. Although there is some evidence of stability of global methylation levels within individuals over time [63], there is likely a degree of misclassification due to this assumption. We focused on LINE-1 repetitive elements in the genome that are often intensively methylated as a proxy for global DNA methylation. Although LINE-1 methylation is strongly correlated with genomic instability, [31,64-66] non-differential misclassification of methylation status due to this proxy measure could have attenuated observed effects in this study. Also, the correlation of 0.66 between methylation levels of the two colon tissue samples indicates some heterogeneity of LINE-1 methylation status in the descending colon and suggests that our results may not be generalizable to tissue biopsies taken from other parts of the colon.

## Conclusions

The study of biomarkers for increased risk of colorectal cancer has enormous potential for understanding colorectal cancer etiology. Our results indicating that LINE-1 DNA hypomethylation in normal appearing colon mucosa is associated with increased adenoma risk, suggest that aberrant global hypomethylation in healthy background colon mucosa represents an underlying pervasive epigenetic aberration, often referred to as a ‘field defect’, which may confer an increased predisposition to the development of colorectal adenomas/cancer [46,67-70]. Though these findings provide some support for a relationship between LINE-1 DNA methylation in colorectal mucosal tissue and adenoma risk, large prospective cohort studies are needed to confirm results. Until such investigations are done, the usefulness of this measure of LINE-1 methylation in colon tissue (or blood leukocyte) DNA as a biomarker of increased adenoma risk that can be used in clinical settings is uncertain. However, even though the clinical significance of our findings is unclear, this study contributes to a better understanding of the role of global DNA methylation in colorectal tissue as an early event in CR carcinogenesis with implications for future CRC etiologic research investigating suspected environmental and lifestyle risk factors using global DNA hypomethylation as an informative end point.

## Competing interests

All authors (Drs. King, Ashbury, Taylor, Tse, Pang, Louw and Vanner) have no conflicts of interest, or financial or other relationships to declare that may influence or bias this work.

## Authors’ contributions

Each author contributed to this manuscript. WK conceived of the study, participated in its design and coordination, drafted the initial manuscript version and performed the statistical analysis. JA coordinated study subject recruitment, implementation and progress of this study, and helped with data interpretation and manuscript organization and editing. ST contributed to the study design, particularly with regards to the choice of methylation measurement method and helped with manuscript editing. YT designed, refined and carried out the high-resolution melt (HRM)-based method for quantifying LINE-1 methylation in participant DNA samples. SP supervised and provided expertise with regards to methylation measurement using HRM and helped to edit the manuscript. JL and SV led the clinical collaborators in terms of recruiting study subjects and contributed to the interpretation of the results in particular with respect to the clinical implications of this study. All authors reviewed and revised the manuscript.

## Pre-publication history

The pre-publication history for this paper can be accessed here:

http://www.biomedcentral.com/1471-2407/14/488/prepub
